# Tuberculosis in Tropical Areas and Immigrants

**DOI:** 10.4084/MJHID.2014.043

**Published:** 2014-06-01

**Authors:** Lorenzo Zammarchi, Filippo Bartalesi, Alessandro Bartoloni

**Affiliations:** 1 Infectious Diseases Unit, Department of Experimental & Clinical Medicine, University of Florence School of Medicine, Florence, Italy; 2 SOD Malattie Infettive e Tropicali, AOU Careggi, Firenze, Italy

## Abstract

About 95% of cases and 98% of deaths due to tuberculosis (TB) occur in tropical countries while, in temperate low incidence countries, a disproportionate portion of TB cases is diagnosed in immigrants.

Urbanization, poverty, poor housing conditions and ventilation, poor nutritional status, low education level, the HIV co-epidemic, the growing impact of chronic conditions such as diabetes are the main determinants of the current TB epidemiology in tropical areas. TB care in these contests is complicated by several barriers such as geographical accessibility, educational, cultural, sociopsychological and gender issues. High quality microbiological and radiological facilities are not widely available, and erratic supply of anti-TB drugs may affect tropical areas from time to time. Nevertheless in recent years, TB control programs reached major achievements in tropical countries as demonstrated by several indicators.

Migrants have a high risk of acquire TB before migration. Moreover, after migration, they are exposed to additional risk factors for acquiring or reactivating TB infection, such as poverty, stressful living conditions, social inequalities, overcrowded housing, malnutrition, substance abuse, and limited access to health care. TB mass screening programs for migrants have been implemented in low endemic countries but present several limitations. Screening programs should not represent a stand-alone intervention, but a component of a wider approach integrated with other healthcare activities to ensure the health of migrants.

## Introduction

Despite encouraging progress, the burden of tuberculosis (TB) remains enormous with about one third of the World population latently infected with the etiologic agent *Mycobacterium tuberculosis*,^1^ 8.7 million new cases of active disease and 1.4 million people died in 2011.^2^ Some authors state that 95% of all cases and 98% of deaths due to TB, occurs in tropical countries.^3^ In the matter of facts among the 22 high burden countries that account for more than 80% of the worldwide incident cases of the disease, 19 have territories geographically located, at least in part, within the tropics (Table 1), indicating tropical areas as the most affected by TB in the World. In high income industrialized countries, the majority of which are located outside the tropics, the overall TB incidence is low, as expected given the inverse correlation between economic development of the country and its TB diffusion.^4^ A disproportionate and growing portion of subjects affected by TB in industrialized countries are migrants from tropical countries,^5^,^6^ configuring this group of subjects as a TB vulnerable population in low endemic areas.

The aim of this review is to give an overview on the historical, epidemiological, clinical and microbiological characteristics and recent control strategies of TB in tropical countries and migrant populations.

## History of TB in Tropical Areas and Migration

Current evidence supports the so called “*Out-of-and-back-to-Africa*” scenario in explaining the origin and global spread of human TB.^7^ Human *M. tuberculosis* complex probably originated in Africa and accompanied the *Out-of-Africa* migrations of modern humans approximately 70,000 years ago.^7^ The three phylogenetically ‘modern’ lineages of *M. tuberculosis* complex (namely the East Asian, the Central Asian/Delhi and the Euro-American lineage) seeded in China, India and Europe, respectively where human population strongly grew during the last few centuries.^7^ As overcrowding conditions and the urbanization increased, TB expanded in these areas and concomitantly spread globally through waves of human migrations.^7^ Through European colonization, the Euro-American lineage of *M. tuberculosis* complex reached other regions such as the Americas in the mid nineteenth century and sub-Saharan Africa at the beginning of the twentieth century.^7^,^8^ Historians and paleopathologists, supported by the detection of mycobacterial DNA in pre-Columbian human remains, suggests that TB was already present in pre-Columbian America. Today most TB in the Americas is caused by the Euro-American lineage, but in the pre-Columbian period, the etiologic agent may have been Asian forms, as would be expected given the original human colonization of the Americas via the Bering Strait. Alternatively, pre-Columbian TB might have been caused by mycobacterial lineages which are now extinct perhaps because they were outcompeted by the Euro-American lineage following the massive influx of Europeans into the Americas between the early eighteenth and early twentieth century.^7^

## Epidemiology and Determinants of Tuberculosis in Tropical Areas

The majority of the known risk factors for acquiring TB infection and for progress to TB disease after the infection are widespread and responsible for the high burden of TB in tropical areas.

About 59% of new estimated TB cases occur in South East Asia and the Western Pacific Regions,^2^ where some of the most populated countries (India, China, Indonesia) and some of the most crowded cities of the World are located.^9^ Urbanization and the consequent overcrowded living conditions, through the increase of shared airspace between individuals, are among the well-known risk factors to acquire TB.^10^

Urbanization is a relatively new, but growing phenomenon in Africa, which is substantially less populated than Asian regions.^9^ However, countries of the African Region account for 26% of the World’s TB cases and they have the highest incidence rate of cases and death *per capita*.^2^ In Africa, the TB epidemic is overlapped and strongly driven by HIV infection which is the most powerful risk factor for developing active TB disease in subjects infected with *M. tuberculosis*.^11^ In this region, 46% of subjects who develop active TB are estimated to be co-infected with HIV (ranging from 8% in Ethiopia to 77% in Swaziland).^2^ In extreme settings, such as gold-mining workforce in South Africa, the annual incidence reaches value of 2,000–3,000 per 100,000 population due to the high rate of HIV co-infection (up to 80% among subjects with active TB) and silicosis.^12^

Concerning countries of the region of Americas, only Brazil, is considered a high burden country given its relevant contribution to the absolute number of TB cases, despite a relatively low overall incidence rate (less than 50 per 100,000 population). However, the burden of TB in Brazil is not uniformly distributed in the national territories with more 70% of cases concentrated in 315 over 5,564 *municípios* corresponding to those hosting the large metropolitan cities where overcrowding and extreme poverty is more frequent.^13^ In some districts of São Paolo (Brazil) where the Human Development Index is particularly low, TB incidence is 167 per 100,000 population.^14^ Some tropical countries such as Haiti, Peru, Bolivia and Suriname have the highest incidence of TB in the Americas (between 100 and 200 per 100,000 population).^15^ HIV co-epidemic is probably one of the most important determinant of the high incidence found in Sub-Saharan Africa, as well in Brazil and Haiti, where about 20% of incident TB cases is HIV co-infected.^15^

Re-infection of subjects with previous latent tuberculosis infection (LTBI), which account for up to 40% of the general population in countries like India,^16^ may play an important role. Even if people with LTBI, have a markedly lower risk of developing TB disease after a re-infection if compared with previously uninfected subjects,^17^ in endemic areas the contribution of re-infection may account up to 70% of TB relapse cases.^18^

A very important, even if distal, determinant of TB in tropical areas is poverty that affects housing conditions, ventilation, nutritional status, education and the access to health care system.^19^ In some areas of India, for example, the amount of monthly earning as well as the schooling degree have been correlated to TB prevalence.^19^ About two third of cases are diagnosed between 15–44 years of age in countries such as South Africa and India. The impact on the health status of young adults in their most economically active years makes that not only does poverty predispose one to TB, but also TB can increase poverty.^19^ In India three to four months of work time, the equivalent to 20–30 per cent of annual household income, are typically lost because of TB.^19^

A growing role of emergent risk factors for progression from latent to active TB, such as certain chronic conditions, have been observed more recently in tropical areas. Smoking doubles the risk of TB and might account for up to half of all deaths in men with TB in India.^20^ Diabetes is associated with an about three-times increase in TB risk accounting for about 20% of smear-positive tuberculosis cases in India in 2000.^20^ Helminthic infestations that are endemic in tropical countries are strongly suspected to negatively impact on TB diseases inducing immunological alterations including alternatively activation of macrophages and Th1-lymphocytes response impairment.^21^ In a cohort of HIV-infected Ugandan adults, *Schistosoma mansoni* infestation was associated with an increased risk of TB progression.^22^ Finally, according to a recent review of the literature on racial difference in susceptibility to infection by *M. tuberculosis*, black skin people may have consistently higher susceptibility to TB if compared to whites skin peoples due to environmental, immunologic, and genetic factors.^23^

## Epidemiology and Determinants of Tuberculosis in Immigrants

TB is a well-known phenomenon linked to migration. By the time of the Italian migration to America between the XX and the XXI century, Italian migrants, resettled in New York city, worked in the factories of the metropolis in very poor housing and living conditions. In this setting, Italian migrants experienced a very high number of TB cases with tens of cases per household and the block where they lived was named “lung’s block”.^24^

**Figure 1:**
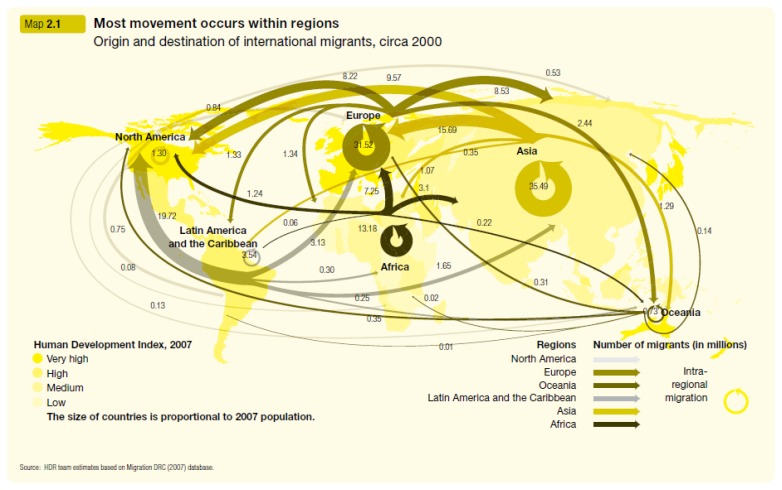
The pattern of inter- and intra-regional migrant movements. [United Nations Development Programme, Summary. Human Development Report 2009. Overcoming barriers: Human mobility and development, United Nations Development Program, (2009). *Reproduced with permission*]

Today, migration is a global social phenomenon that may be defined as a movement of people within and among countries as a consequence of wealth disparity, poverty, wars, natural disasters and political persecution.^19^,^25^ To date there are an estimated 740 million internal migrants and 200 million of international migrants (Figure 1),^26^ without considering irregular migrants of which it is difficult to make an affordable estimate.

Many migrants originate from countries where TB have a high incidence, such as tropical countries, and resettle in higher income countries, such as Unites States, Canada, Australia, New Zealand and western Europe, where TB incidence is now very low (less than 10 per 100,000 population) (Table 2).^25^

In the United States (US), TB cases in foreign-born persons accounted for 62% of total TB cases in 2011 with Asians accounting for 29% and Hispanics/Latinos for 21% of all cases.^6^ Considering the countries belonging to the European Economic Area (EEA), foreign origin persons represented 26% of cases diagnosed in 2011.^5^ However, this percentage rises to more than 40% in the western European countries holding the highest proportions of migrants in Europe (Table 2).^5^ In the EEA the majority of foreign origin subjects diagnosed with TB in 2009 originated from Asia (34.2%), Africa (28.6%) and other European countries (19.9%).^27^

Immigration is playing an important role in the epidemiology of TB in certain high burden countries with emerging economies. In some districts of São Paolo (Brazil), the portion of TB cases diagnosed in Bolivian migrants grew up to 53% of total cases in the period 1998–2008,^14^ while migrant workers from rural areas of China resettled in the district of Shanghai accounted for 67.4% of cases diagnosed in 2006–2008.^28^

It is clear that migrants currently play an important role in determining the current epidemiology of TB in countries where they settled. However reports from different high income countries with well-performing screening and treatment systems have shown that foreign-born TB patients do not contribute importantly to TB transmission in the native population.^25^,^29^,^30^ Based on genotyping analysis, a variable portion of TB cases in native populations (ranging from 2% to 17%) has been attributed to transmission from foreign-born subjects.^31^,^32^ In more recent study, performed in Denmark, transmission from Danes to migrants occurred 2.5 times more frequently than vice-versa.^30^

Migrants are exposed in their country of origin to several risk factors for TB infection and progression as already explained in the above paragraph.

The incidence in the countries of origin is the strongest predictor of TB incidence in migrants according to some authors.^33^ However in other studies the TB incidence in selected migrant communities was found to be lower or higher if compared with the incidence in the country of origin according to the degree of socio-economical integration of the community.^34^,^35^ After migration, foreign born people are exposed to a series of additional factors that have been associated with an increasing risk of acquiring or reactivating TB infection such as poverty, stressful living condition, material deprivation, social inequalities, unemployment, fewer educational opportunities, overcrowded housing, malnutrition, substance abuse, and limited access to health care.^36^

TB in migrants may occur as a consequence of a reactivation of a LTBI acquired in the country of origin, but also because of a new infection acquired in the host country after resettlement or during travel in the country of origin. Molecular epidemiology studies have helped to understand the relevance of LTBI reactivation in the pathogenesis of TB in migrants. In these studies, clustered cases (defined as two or more cases with clonally related TB strains) are assumed to belong to a chain of recent transmission, while cases whose *M. tuberculosis* isolates display unique patterns are regarded as sporadic and assumed to be caused by reactivation.^35^ According to the different studies, 10%-45% of TB cases diagnosed in foreign-born patients are clustered,^35^,^37^,^38^ this means that a relevant proportion of active TB cases is probably caused in immigrants by new infection acquired after migration, even if the majority of cases are due to LTBI reactivation acquired before migration.

As well known a considerable portion (23–53%) of TB cases in migrants is diagnosed in the first years (2–4) after resettlement in the host country.^6^,^39^–^41^ However, the reasons for this phenomenon are not completely clear. Some authors suggest that the stressful and socioeconomically disadvantaged living conditions in the first years after migration could contribute to the reactivation of TB early after arrival.^36^ However, the risk of TB in migrants was found to persist for their life time.^42^ For example, in one study, one third of TB cases in Australia migrants were diagnosed 10 years after arrival, and this interval was larger when considering European migrants only.^43^

An increased risk of TB is still present in second generation migrants in which a link to endemic countries persists after migration through social networks or travel in the country of origin of their ethnic minority group.^44^,^45^ In United Kingdom (UK), for example, the highest incidence rates in UK born subjects are in ethnic minority groups.^46^ The role of travel to visit friends and relatives on the risk of TB infection during an international travel is not exactly known. However the risk for an international traveler approximates the risk of transmission in the local population of the country of destination,^47^ and it is associated with duration of travel.^48^ Among travelers, immigrant visiting friends and relatives, especially children, are likely to represent a group at higher risk, perhaps due to their closer contact with the local population as shown by several studies that report an association between TST positivity and return to the country of origin.^49^

## TB Diagnosis and Management in Tropical Areas

The most common symptom of pulmonary TB is a productive cough for more than 2 weeks, which may be accompanied by other respiratory symptoms (shortness of breath, chest pains, hemoptysis) and/or constitutional symptoms (loss of appetite, weight loss, fever, night sweats, and fatigue).^50^ The presence of those symptoms are enough to met the definition of suspected TB case according to the World Health Organization (WHO).^50^

For a patient living in a remote tropical village that has cough for more than 2 weeks, the way to achieve the correct diagnosis of TB, to start anti-tubercular treatment and to complete it successfully may be very long and full of hurdles. According to a systematic review, in resource limited countries the average patient delay (time from the onset of symptoms until the patient see the first health care practices) and average health system delay (time from the first health care seeking for diagnosis until the diagnosis made) are 31.7 days and 28.5 days, respectively.^51^

Low educational level, low awareness and knowledge about TB and sociopsychological barriers, gender inequalities, are the first bottlenecks for the initial health access.^52^ Believing TB incurable or caused by evil spirit, possible social exclusion following the diagnosis of TB (stigma), fear of revealing HIV status to neighbors, since TB is closely related to HIV in tropical areas, are some the factors conditioning health seeking behaviors and the diagnostic delay in tropical countries.^52^

Rural residence and other geographical barriers are further limiting factors in the diagnostic path. Ideally a health facility able to start the clinical management of a suspected TB case should be within 1-day walking distance as many patients have limited access to motorized vehicles.^52^ However, in 2011 the WHO estimates that only 15 of the 22 high TB burden countries met the target of having 1 microscopy centre per 100,000 population and 17 of the 36 countries with a high burden of TB and multidrug-resistant (MDR) TB have the recommended capacity of 1 laboratory to perform culture and drug susceptibility test (DST) per 5 million population.^2^

Initial visit to a governmental low-level healthcare facility, initial visit to traditional or unqualified practitioner or even a visit to a private practitioner are factors associated with further diagnostic delay.^52^ The delay in diagnosis from this point forward reflects a lack of effective diagnostic tools and follow-up routines since a correct diagnosis requires both good training and available diagnostic facilities.^52^ Table 3 reports the list of the most important factors associated with diagnostic delay according to a systematic review.^52^

Diagnosis of TB is a challenge not only in tropical countries, but anywhere resources are limited. Conventional microbiological methodology such as direct microscopy and culture, when available, have intrinsic limitation that have constrained TB care and control up to now.^2^ Smear microscopy has a low sensitivity (about 64%),^53^ which is even lower in HIV positive patients^54^ and in children.^55^ Culture is considered the gold standard but requires some weeks to give a positive result and even new liquid culture techniques, which are more sensitive and allow a faster grown of mycobacteria, are seldom available in resource-constrained settings largely because of cost.^56^ Radiology has an important role in the diagnosis of TB but the equipment is expensive to obtain, maintain, operate and experienced radiologist are required in order to interpret the often non-specific radiological signs of TB.^3^ Few years ago, the situation of radiological manpower and facilities in sub-Saharan Africa was reported to show a desperate shortage of radiologists, radiographers and equipment, with most of services located in the capital with few at rural hospital and CT scanners or high resolution ultrasound machines available only in 40% of these countries.^57^

In view of the paucity of diagnostic tools available, the challenge of TB diagnosis in the tropics may be related to problems of differential diagnosis. In the tropics, pulmonary TB must be distinguished from other rare endemic and ubiquitous conditions such bacterial pneumonia, histoplasmosis, paracoccidioidomycosis, coccidioidomycosis, melioidosis, actinomycosis, paragonimiasis, echinococcosis, nocardial and aspergillus mycetoma, dirofilariosis, neoplasm, sarcoidosis,^58^,^59^ which could be a hard task, given the limited diagnostic resources available.

Hopefully, the recent availability of new rapid tests could revolutionize TB care in endemic and tropical countries. The new test Xpert MTB/RIF, which has been endorsed by WHO in December 2010, is a cartridge-based automated diagnostic test that has three main advantages if compared with older tests: 1) it enables simultaneous detection of *M. tuberculosis* complex and rifampicin-resistant associated genotype; 2) provides accurate results in less than two hours so that patients can be offered proper treatment on the same day; 3) has minimal bio-safety requirements, training, and can be housed in non-conventional laboratories.^60^ According to a meta-analysis, the pooled sensitivity was 98.7% for pulmonary sputum positive TB and 75% for sputum negative TB with an overall specificity of 98.4%, while the sensitivity on non-respiratory clinical samples resulted to be 80.4%.^61^, ^62^ Xpert MTB/RIF showed dramatic cut of the time needed to start treatment, especially in smear negative cases, and to obtain rifampicin susceptibility result.^63^ With the introduction of Xpert MTB/RIF, there has been also an increase of the number of microbiologically confirmed TB in children,^62^ and an increase of the number of pulmonary TB cases detected in HIV positive patients when compared with microscopy.^62^ Between its endorsement by WHO and the end of June 2012, 1.1 million test cartridges were procured in 67 (46%) of the 145 countries eligible to purchase them at initial concessional prices (9.98 $ per test from August 2012).^62^ Currently, WHO strongly recommends the use of Xpert MTB/RIF for use, as the primary diagnostic test, in individuals suspected of having MDR or HIV-associated TB and in testing cerebrospinal fluid specimens from patients presumed to have TB meningitis; furthermore, WHO provides “conditional recommendations” for its use in other settings.^64^ However, several weakness of this new tool have already been highlighted, including elevated cost of the platform (17,000$), the sophisticated hardware needing calibration and maintenance, need of continuous electrical power supply and air conditioning, short shelf life of cartridges needing good procurement system, need for cartridges storage at 2–28°C and system for disposal after use.^62^ Concerning other relatively recent diagnostic tools such as interferon gamma release assays (IGRA) and serological test for TB, WHO recommended against their use in middle and low income countries for the diagnosis of both active and LTBI.^2^

Directly Observed Treatment (DOT) of TB reduces TB related death, disability and transmission, and it is highly cost-effective intervention even in the lowest income countries.^2^ Treatment of a drug-sensitive TB, case, takes 6 months, while treatment for MDR TB case takes 18–20 months according to the WHO recommendations.^2^ The target of 85% of treatment success for new TB cases has been achieved at global level, but it is still under the goal threshold in African (73%), Americas (74%) and European Regions (74%), with the lowest rate (53%, possibly underestimated) reached by South Africa.^2^

Concerning patients with MDR-TB, that represent a growing portion of cases, only 44% to 58% completed treatment successfully according to different Regions.^2^ In Africa, 19% of patients with MDR-TB is not able to complete the treatment because of death.^2^

TB and HIV are strictly related, and the management of the two conditions must go hand in hand. To date only 40% of patients with TB are tested for HIV, with the African Region performing better than all other regions (69%).^2^ However only 56% of people eligible for antiretroviral therapy is receiving it in Africa.^65^ The assessment of the HIV status in a patient with TB is essential since the timely start of antiretroviral therapy has been demonstrated to reduce significantly the mortality of the patient.^66^–^68^ Treatment success of TB is hampered by several problems that may be amplified especially in tropical areas, such as problematic access to health care facilities, poor adherence to treatment, availability of quality drugs, high rate of MDR cases, and HIV co-infection. Treatment default implies persistence of infection source, increased mortality, increased relapse rates and increased risk of the development of resistant strains.

In different case control studies, frequently identified risk factors associated with a default of the patients under TB treatment in tropical areas were inadequate knowledge on TB,^69^,^70^ illiteracy or low education level,^70^,^71^ herbal medication use,^69^ low income,^69^ alcohol abuse,^69^–^71^ HIV co-infection,^69^,^71^ male gender^69^ poor patient-provider interaction,^70^ side effects to anti TB drugs.^70^

The erratic supply of drugs that may affect some areas is another relevant problem. A survey carried out in Ethiopia in 2008 showed that the first line drugs for TB treatment were not available in about 20% of 48 health facilities that were supposed to have.^72^ Doctors without Borders recently reported a drug supply crisis in Mthatha (South Africa) started in 2013.^73^ During a survey done in May 2013 in the area, still 40% of facilities suffered stock-outs of antiretroviral drugs and/or TB drugs with a median duration for reported stock-outs of 45 days.^73^

## TB Diagnosis and Management in Immigrants

The access to health system, including TB diagnostic and treatment services is lower in migrant populations compared to native subjects. Migrants have a longer patient diagnostic delay for TB (defined as the time elapsed from the onset of symptoms and the first medical consultation), while natives have a longer health care diagnostic delay (defined as the time elapsed between the first medical consultation and the initiation of treatment).^74^,^75^ The increased patient delay is possibly due to a combination of reasons that hinder migrants of using the available TB services. Among those factors, there are language barriers, possible lack of medical insurance, fear of deportation (for illegal migrants) or discontinuation of their employment^74^,^75^ and competing socio-economic priorities may prevail over health issues. Even if in most of countries TB diagnosis and treatment are provided for free at government health facilities to all migrants, including illegal migrants, additional costs of transport and the time needed to perform medical consultations may represent significant obstacles for access to health system for migrants on low wages.^76^ The longer health care diagnostic delay in native subjects can be explained by the lower TB incidence among native subjects in low endemic countries, that leads physicians to reduce their index of suspicion regarding the possibility of TB diagnosis and ordering other tests rather than TB-diagnostic tests.^74^,^75^

TB treatment in migrant populations can be challenging due to lower adherence to treatment.^77^–^79^ According to a recent study, loss to follow-up in TB cases in UK appears to occur primarily in young male adults and in subjects born outside the UK, particularly those who migrated within the 2 years prior to diagnosis.^77^ Moreover, this study showed that lost to follow-up patients were more frequently infected with a resistant *M. tuberculosis* strain compared to patients who completed or were still on treatment (11% vs. 7.4%), highlighting the vicious circle among poor compliance to treatment and resistance to antitubercular drugs.^77^ Higher therapeutic abandonment has been recently found also in foreign born patients if compared to natives in Granada (Spain)^79^ and in Chinese internal migrants if compared to permanent residents.^78^

Another challenging issue in the management of TB in migrants, in low endemic countries, is the high frequency of MDR-TB in this population if compared to natives (Table 4). The majority of European and other low prevalence countries, excluding some of the high priority countries in the WHO European Region (such as Latvia, Lithuania, Bulgaria and Estonia), report higher prevalence of MDR-TB cases in migrants if compared to the native population.^80^ This is probably due to the high prevalence of MDR-TB in migrants countries of origin and possibly to the low compliance to treatment that characterizes migrants and leads to acquire drug resistance.

Given the epidemiological importance of migrant subjects in determining the epidemiology of TB in industrialized countries, many of those countries implemented different control measures for TB, including mass screening programs. The rationale of these programs is the early detection and treatment of active and then contagious TB cases, in order to prevent *M. tuberculosis* transmission within the host country.^81^ Indeed screening for active TB may decrease the period of infectiousness by as much as 33%.^82^ Secondary benefits of immigration screening are reduced transmission of TB in the country of origin and during travel.^81^

A recent survey showed that high-income industrialized countries have widely different approaches to the screening of migrants arriving in their territories.^83^ In the majority (23 of 25, 92%) of cases, screening is performed after the arrival, while only 36% (9 of 25) and 20% (5 of 25) of countries perform also pre-arrival and at-arrival screening respectively, according to the different type of immigrants.^83^ The majority of countries (25, 86.2%) screens for active TB and most commonly (76% of cases) the screening is compulsory.^83^ The most commonly used tool for screening for active TB in adult migrants is a chest radiograph, which is used by 22 of 25 (88%) industrialized countries alone or in combination with clinical examination and less commonly, with tuberculin skin test (TST).^83^ However, screening protocols based on chest x-ray only are unable to detect cases of extrapulmonary TB, which represent a not negligible portion of TB cases in migrant patients (24% of cases diagnosed in non US born patient in US).^84^ Moreover, concerning pulmonary TB, chest x-ray shows a sensitivity of 86–97% and a specificity of 75–89% according to the different criteria used for imaging interpretation.^85^ The median yield of screening for TB disease (portion of patients with active TB among those screened) has been found quite low (0.18%) in the EEA.^86^

Only 16 of 29 (55.2%) countries, inquired in the above survey, screens for LTBI by using TST in 68.8% of cases or TST plus a confirmatory IGRA in 25% or an IGRA alone in 18.8%.^83^ Some authors strongly support the implementation of screening for LTBI based on the evidence that the majority of active TB cases, diagnosed in migrants, are due to a LTBI reactivation acquired in the country of origin^38^ and on the findings of cost-effectiveness analysis.^87^,^88^ According to a cost-effectiveness study, the most suitable strategy would be to screen with an IGRA (in particular QuantiFERON-TB Gold In-Tube. Carnegie, Cellestis, Australia) test all migrants coming in UK from countries with an incidence of more than 250 cases per 100,000 (incremental cost-effectiveness ratio [ICER] of £ 21,565 per prevented case of TB) without port of arrival chest x-ray.^88^ However these results are opposite to those of a previous study done in Canada that found chest radiograph the most and Quantiferon the least cost-effective strategy to screen migrants.^89^ The discordance of the findings are probably explained by the different assumptions done in the models such as high rates of acceptance and completion of chemoprophylaxis assumed in the English study and low prevalence of latent infection in new immigrants assumed in the Canadian study.

While the attention of the different governmental programs and several scientists seems to focus on mass screening programs for active TB and/or LTBI, this kind of interventions should not represent a stand-alone intervention, but a component of a wider approach.^86^ The six points proposed by the STOP TB strategy (Table 5),^80^ which address the activities to deal with TB at global level, could be a useful paradigm for drawing a more comprehensive approach for TB control in migrant populations.

Implementing DOT in low endemic areas or newer socially and culturally acceptable programs to sustain treatment adherence, address MDR-TB, contributing to health systems strengthening with the presence of peer educators and culturally-oriented health staff, engaging all care providers including members of Non Governmental Organizations and voluntary associations, empowering migrants communities and promoting research to find new possible operational solutions and tools for TB care and prevention could likely be of benefit for future TB control programs in migrants.^90^

In conclusion TB care should be offered and integrated with other healthcare activities within the context of a holistic approach to ensure the health and wellbeing of new entrant migrants.^76^,^91^,^92^

## Figures and Tables

**Table 1:** High burden countries with territories located within the tropics and estimated incidence.^2^

High burden country in the tropics	Estimated incidence (rate per 100,000 population)	Estimated number of cases (number in thousands)	Estimated portion respect to the global burden
Bangladesh	225	340	4%
Brazil	42	83	1%
Cambodia	424	61	0.7%
China	75	1000	11.5%
Democratic Republic of the Congo	327	220	2.5%
Ethiopia	258	220	2.5%
India	181	2200	25%
Indonesia	187	450	5.2%
Kenya	288	120	1.4%
Mozambique	548	130	1.5%
Myanmar	381	180	2%
Nigeria	118	190	2.2%
Philippines	270	260	3%
South Africa	993	500	5.7%
Thailand	124	86	1%
Uganda	193	67	0.8%
United Republic of Tanzania	169	78	0.9%
Viet Nam	199	180	2%
Zimbabwe	603	77	0.9%

Afghanistan, Pakistan and Russian Federation are considered high burden countries but they have not territories located within the tropics.

**Table 2:** Number and portion of cases of active tuberculosis in foreign origin people diagnosed in countries of the European Economic Area and selected low TB incidence countries.^5^,^6^,^39^,^93^,^94^

Country	Number of TB cases diagnosed in foreign origin subjects	Portion of TB cases diagnosed in foreign origin subjects
European Economic Area*		
Austria	326	47.5%
Belgium	544	52.1%
Bulgaria	9	0.4%
Cyprus	45	83.3%
Czech Republic	112	18.7%
Denmark	235	61.7%
Estonia	48	14.1%
Finland	79	24.3%
France	2,456	49.7%
Germany	2,025	46.9%
Greece	216	44.2%
Hungary	27	1.9%
Ireland	179	42.1%
Italy	1,677	47.6%
Latvia	59	6.7%
Lithuania	44	2.3%
Luxemburg	21	80.8%
Malta	28	84.8%
Netherland	710	70.5%
Poland	38	0.4%
Portugal	385	15.2%
Romania	50	0.3%
Slovakia	3	0.8%
Slovenia	57	29.7%
Spain	2,138	31.6%
Sweden	524	89.4%
United Kingdom	6,287	70.1%
Iceland	7	77.8%
Liechtenstain	-	-
Norway	317	87.8%
United States*	6,510	62%
**Canada**°	~1,040	66%
**Australia**^	1,141	88%
New Zealand*	227	75.4%

* Data referred to 2011

° Data referred to 2010;

^ Data referred to 2009

**Table 3:** Risk factors for TB diagnostic delay (adapted from Storla DG et al).^52^

Coexistence of chronic cough and/or other lung diseases
Negative sputum smear
Extrapulmonary TB
Rural residence
Low access to healthcare (geographical or socio-psychological barriers)
Initial visit to government low-level healthcare facility
Initial visit to traditional or unqualified practitioner
Initial visit to private practitioner
Initial visit to tertiary-level services/hospital
Old age
Poverty
Female sex
Alcoholism or substance abuse
History of immigration
Low educational level and/
or low awareness and knowledge about TB
Generally poor health
Smoking
Coexistence of sexually transmitted diseases
Less severe and indifferent symptoms
No hemoptysis
Married
Single
Large family size
Farmer
White (vs. aboriginal)
Muslim
Belonging to an indigenous group
No insurance
Beliefs about TB (not curable, caused by evil spirits, etc.)
Stigma
Self-treatment

The study by Storla DG et al. was a systematic review that includes 58 articles in the final analysis. Thirty eight studies (65%) were carried out in countries with incidence >40 per 100,000 population (the majority of which tropical), while 20 studies (35%) were carried out in non-tropical lower incidence countries.

“HIV” and “Initial visit to tertiary-level services/hospital” have been removed from the original table because they were negatively associated with diagnostic delay according to the majority of the studies.

**Table 4:** Prevalence of Multi Drug Resistance in native and foreign origin subjects diagnosed with tuberculosis in countries of the European Economic Area and selected low TB incidence countries.^5^,^6^,^39^,^93^,^94^

Country	MDR prevalence in subject with TB (%)	
	Native	Foreign origin
European Economic Area*		
Austria	0	8.9
Belgium	0	3.7
Bulgaria	7.5	0
Cyprus	0	3
Czech Republic	0.6	6.2
Denmark	0	1.8
Estonia	30.3	25.8
Finland	1.3	4.4
France	-	-
Germany	0.6	3.4
Greece	0	5.6
Hungary	1.3	10
Ireland	0	1.7
Italy	1.4	4.2
Latvia	14.9	13.3
Lithuania	20.9	29.4
Luxemburg	0	13.3
Malta	0	0
Netherland	0	2.8
Poland	0.8	3.8
Portugal	1.1	5.4
Romania	8.8	14.3
Slovakia	1.6	0
Slovenia	0	0
Spain	-	-
Sweden	0	3.9
United Kingdom	0.3	2.1
Iceland	-	0
Liechtenstain	-	-
Norway	0	1.7
United States*#	0.6	1.7
**Canada**	-	-
**Australia**	-	-
New Zealand*	0	1.1

**Footnotes:**

* Data referred to 2011;

# Data referred to cases without previous diagnosis of TB.

**Table 5:** The six components of the STOP TB strategy.^80^

1) Pursue high-quality DOTS expansion and enhancement
2) Address TB-HIV, MDR-TB, and the needs of poor and vulnerable populations
3) Contribute to health system strengthening based on primary health care
4) Engage all care providers
5) Empower people with TB, and communities through partnership
6) Enable and promote research
